# Supplementary material for: Identification and Patient Benefit Evaluation of Machine Learning Models for Predicting 90-Day Mortality After Endovascular Thrombectomy Based on Routinely Ready Clinical Information
Source: Bioengineering (Basel). 2025 Apr 28;12(5):468. doi: 10.3390/bioengineering12050468 (PMC12109170; doi:10.3390/bioengineering12050468)
Supplement: Supplementary file 1 [file bioengineering-12-00468-s001.zip › bioengineering-3581346-supplementary.pdf]

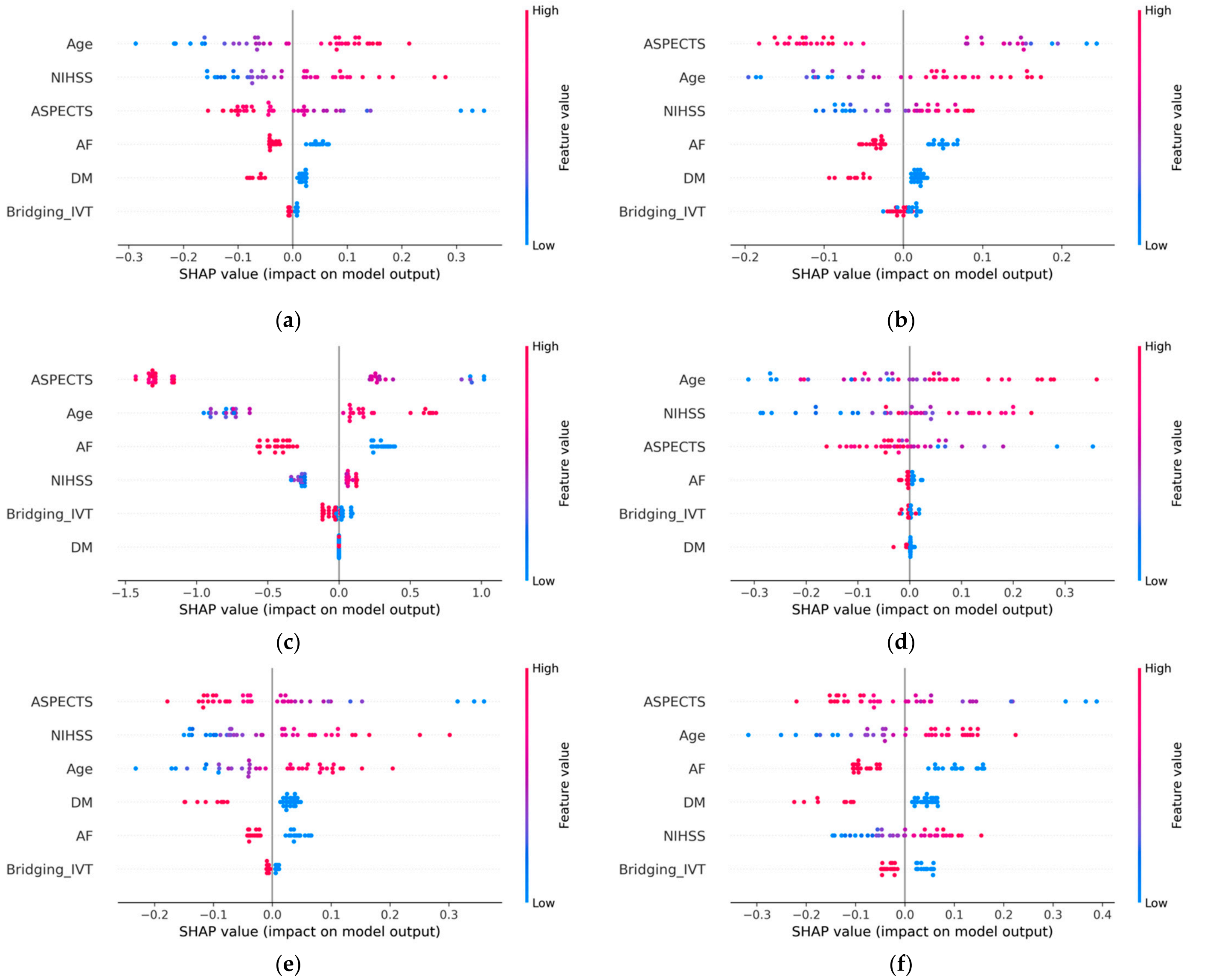

Figure S1. Plot importance information in model I (a) LR, (b) RF, (c) XGB, (d) KNN, (e) SVM, and (f) NN. KNN: K-Nearest Neighbor; LR: Logistic Regression; NN: Neural Network; RF: Random Forest; SVM: Support Vector Machine; XGB: Extreme Gradient Boosting.

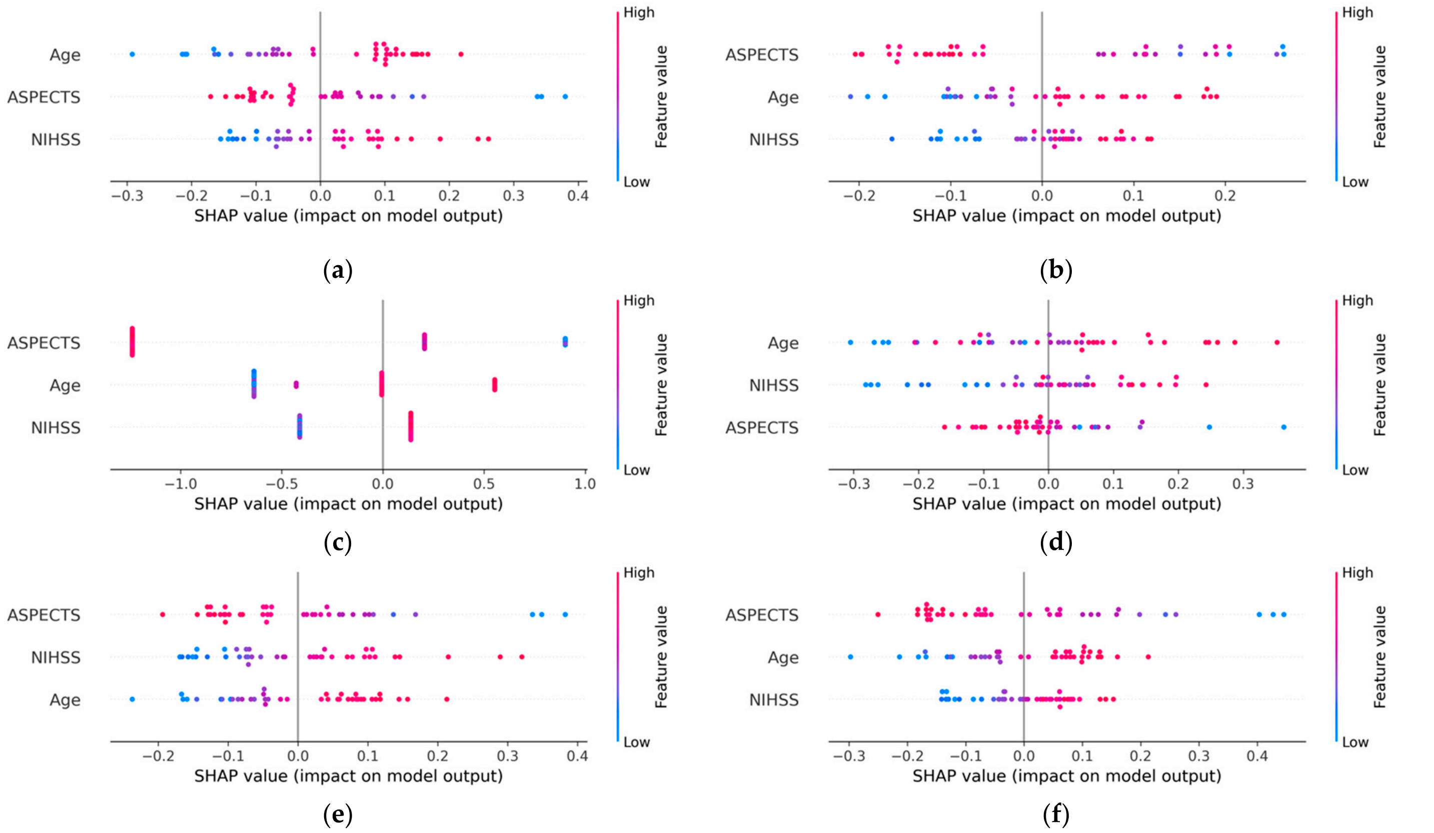

Figure S2. Plot importance information in model II (a) LR, (b) RF, (c) XGB, (d) KNN, (e) SVM, and (f) NN. KNN: K-Nearest Neighbor; LR: Logistic Regression; NN: Neural Network; RF: Random Forest; SVM: Support Vector Machine; XGB: Extreme Gradient Boosting.

Table S1. Calculation of prediction score

Scores (Maximum)	Calculation
HIAT2	Age: <60 = 0; 60-79 = 2; ≥80 = 4;
(10)	Baseline NIHSS: ≤10 = 0; 11-20 = 1; ≥21 = 2;
	Admission Glucose Level: <150 mg/dL = 0; ≥150 mg/dL = 1;
	ASPECTS: 8-10 = 0; ≤7 = 3
THRIVE	Age: ≤59 = 0; 60-79 = 2; ≥80 = 4;
(9)	Baseline NIHSS: ≤10 = 0; 11-20 = 1; ≥21 = 2;
	Chronic Disease Scale: Diabetes mellitus = 1; Atrial fibrillation = 1; Hypertension = 1
PRACTICE	Age: <80 = 0; ≥80 = 2;
(6)	Previous stroke = 1;
	Occlusion site: MCA = 0; ICA/BA = 2;
	Admission NIHSS: <18; = 0; ≥18 = 1

ASPECTS: Alberta Stroke Program Early CT Score; HIAT2: Houston Intra-Arterial recanalization 2; NIHSS: National Institutes of Health Stroke Scale; PRACTICE: Predicting 90-days mortality of AIS with MT; THRIVE: Totaled Health Risks In Vascular Events.

Table S2. Demographic and clinical characteristics between patients with and without 90-day mortality in external validation data set

Variables	Overall (n = 312)	90-day mortality (n = 56)	No 90-day mortality (n = 256)	P-value
Age, years, median (IQR)	72 (63.75-79)	76.5 (68.5-83)	71 (62-78.25)	**0.02**
Male, n (%)	186 (59.6)	29 (51.8)	157 (61.3)	0.187
Baseline NIHSS, median (IQR)	14 (11-18)	17 (13-22)	13 (10-17.25)	**<0.001**
Admission SBP, mmHg, mean (SD)	138.02 (23.24)	138.11 (23.24)	138.00 (23.29)	0.976
Admission blood glucose level, median (IQR)	6.47 (5.31-8.09)	8.00 (6.08-9.85)	6.33 (5.20-7.62)	**<0.001**
History of previous stroke, n (%)	67 (21.5)	13 (23.2)	54 (21.1)	0.726
Atrial Fibrillation, n (%)	99 (31.7)	24 (42.9)	75 (29.3)	**0.048**
Hypertension, n (%)	236 (75.6)	46 (82.1)	190 (74.2)	0.211
Diabetes Mellitus, n (%)	101 (32.4)	22 (39.3)	79 (30.9)	0.222
ASPECTS, median (IQR)	5 (4-7)	4 (3-6)	5 (4-7)	**0.002**
ICA/BA Occlusion, n (%)	97 (31.1)	20 (35.7)	77 (30.1)	0.409
Bridging IVT, n (%)	138 (44.2)	22 (39.3)	116 (45.3)	0.411

ASPECTS: Alberta Stroke Program Early CT Score; BA: Basilar Artery; ICA: Internal Carotid Artery; IQR: Interquartile Range; IVT: Intravenous Thrombolysis; NIHSS: National Institute of Health Stroke Scale; SD: Standard Deviation.

Table S3. Demographic and clinical characteristics between the training set and test set

Variables	Training set (n = 111)	Test set (n = 40)	P-value
Age, years, median (IQR)	78 (69.5-85)	79 (69-85)	0.879
Male, n (%)	41 (36.9)	16 (40)	0.732
Baseline NIHSS, median (IQR)	24 (19-27.5)	22 (17.75-27)	0.611
Admission SBP, mmHg, mean (SD)	155.41 (26.78)	160.4 (30.94)	0.334
Admission blood glucose level, median (IQR)	7.1 (6.15-8.7)	7.5 (6.2-10.25)	0.234
History of previous stroke, n (%)	28 (25.2)	6 (15)	0.184
Atrial Fibrillation, n (%)	55 (49.5)	23 (57.5)	0.388
Hypertension, n (%)	79 (71.2)	23 (57.5)	0.113
Diabetes Mellitus, n (%)	28 (25.2)	9 (22.5)	0.731
ASPECTS, median (IQR)	8 (6-9)	8 (6-9)	0.729
ICA/BA Occlusion, n (%)	42 (37.8)	15 (37.5)	0.970
Bridging IVT, n (%)	50 (45.0)	22 (55)	0.280

ASPECTS: Alberta Stroke Program Early CT Score; BA: Basilar Artery; ICA: Internal Carotid Artery; IQR: Interquartile Range; IVT: Intravenous Thrombolysis; NIHSS: National Institute of Health Stroke Scale; SD: Standard Deviation.

Table S4. Hyperparameters utilized and the final selected hyperparameters for all algorithms used in model I

Algorithms	Hyperparameters name	Hyperparameters value	Selected Hyperparameters
LR	'C'	[0.1, 1, 10]	0.1
	'solver'	['newton-cg', 'lbfgs', 'liblinear', 'sag', 'saga']	'newton-cg'
RF	'n_estimators'	range (0, 200, 10)	80
	'max_features'	['auto', 'sqrt', 'log2']	'log2'
	'max_depth'	range (0, 20, 1)	14
	'criterion'	['gini', 'entropy']	'entropy'
	'min_samples_split'	range (0, 20, 1)	14
	'min_samples_leaf'	range (0, 20, 1)	7
XGB	'learning_rate'	[0.1, 0.01, 0.001]	0.1
	'min_child_weight'	range (0, 10, 1)	8
	'n_estimators'	range (0, 200, 10)	70
	'gamma'	[0, 0.5, 1, 1.5, 2, 5]	2
	'subsample'	[0.7, 0.8, 0.9, 1.0]	1.0
	'colsample_bytree'	[0.3, 0.4, 0.5, 0.6, 0.7, 0.8]	0.6
	'max_depth'	range (0, 10, 1)	4
KNN	'n_neighbors'	np.arange(2, 30, 1)	8
SVM	'C'	[0.1, 0.01, 0.001, 1]	0.001
	'kernel'	['linear', 'rbf', 'poly']	'poly'
	'gamma'	[0.1, 0.01, 0.001]	0.01
NN	'activation'	['relu', 'logistic']	'logistic'
	'hidden_layer_sizes'	[[90, 180, 90], [90, 120, 90], [90, 90], [90, 180]]	[90, 90]
	'alpha'	[0.01, 0.001, 0.0001]	0.001
	'batch_size'	[32,64]	64
	'learning_rate_init'	[0.01, 0.001]	0.001
	'solver'	[''adam'']	'adam'

KNN: K-Nearest Neighbor; LR: Logistic Regression; NN: Neural Network; RF: Random Forest; SVM: Support Vector Machine; XGB: Extreme Gradient Boosting

Table S5. Hyperparameters utilized and the final selected hyperparameters for all algorithms used in model II

Algorithms	Hyperparameters name	Hyperparameters value	Selected Hyperparameters
LR	'C'	[0.1, 1, 10]	0.1
	'solver'	['newton-cg', 'lbfgs', 'liblinear', 'sag', 'saga']	'newton-cg'
RF	'n_estimators'	range (0, 200, 10)	110
	'max_features'	['auto', 'sqrt', 'log2']	'sqrt'
	'max_depth'	range (0, 10, 1)	6
	'criterion'	['gini', 'entropy']	'entropy'
	'min_samples_split'	range (0, 10, 1)	2
	'min_samples_leaf'	range (0, 10, 1)	6
XGB	'learning_rate'	[0.1, 0.01, 0.001]	0.1
	'min_child_weight'	range (0, 10, 1)	8
	'n_estimators'	range (0, 200, 10)	70
	'gamma'	[0, 0.5, 1, 1.5, 2, 5]	2
	'subsample'	[0.7, 0.8, 0.9, 1.0]	1.0
	'colsample_bytree'	[0.3, 0.4, 0.5, 0.6, 0.7, 0.8]	0.6
	'max_depth'	range (0, 10, 1)	4
KNN	'n_neighbors'	np.arange(2, 30, 1)	8
SVM	'C'	[0.1, 0.01, 0.001, 1]	0.001
	'kernel'	['linear', 'rbf', 'poly']	'poly'
	'gamma'	[0.1, 0.01, 0.001]	0.01
NN	'activation'	['relu', 'logistic']	'logistic'
	'hidden_layer_sizes'	[[90, 180, 90], [90, 120, 90], [90, 90], [90, 180]]	[90, 90]
	'alpha'	[0.01, 0.001, 0.0001]	0.0001
	'batch_size'	[32,64]	32
	'learning_rate_init'	[0.01, 0.001]	0.001
	'solver'	[''adam'']	'adam'

KNN: K-Nearest Neighbor; LR: Logistic Regression; NN: Neural Network; RF: Random Forest; SVM: Support Vector Machine; XGB: Extreme Gradient Boosting

Table S6. P-value for comparing the AUC of algorithms between models I and II in test set

Algorithms	Model I	Model II	P-Value
LR	0.708	0.730	0.5010
RF	0.690	0.693	0.9471
XGB	0.705	0.693	0.6629
KNN	0.734	0.744	0.6587
SVM	0.690	0.708	0.6668
NN	0.675	0.725	0.3899

KNN: K-Nearest Neighbor; LR: Logistic Regression; NN: Neural Network; RF: Random Forest; SVM: Support Vector Machine; XGB: Extreme Gradient Boosting
